# Insomnia is associated with road accidents. Further evidence from a study on truck drivers.

**DOI:** 10.1371/journal.pone.0187256

**Published:** 2017-10-31

**Authors:** Sergio Garbarino, Nicola Magnavita, Ottavia Guglielmi, Michelangelo Maestri, Guglielmo Dini, Francesca Maria Bersi, Alessandra Toletone, Carlo Chiorri, Paolo Durando

**Affiliations:** 1 Department of Neuroscience, Rehabilitation, Ophthalmology, Genetics and Maternal/Child Sciences (DINOGMI), University of Genoa, Genoa, Italy; 2 Department of Health Sciences, Postgraduate School in Occupational Medicine, University of Genoa, Genoa, Italy; 3 Occupational Health Unit, Institute of Public Health, Università Cattolica del Sacro Cuore, Rome, Italy; 4 Neurology Unit, Department of Clinical and Experimental Medicine, University of Pisa, Pisa, Italy; 5 Occupational Medicine Unit, Ospedale Policlinico San Martino, Genoa, Italy; 6 Department of Educational Sciences, University of Genoa, Genoa, Italy; University of Rome Tor Vergata, ITALY

## Abstract

**Background:**

Sleep-related problems are known risk factors for road accidents. However, very few studies have investigated the role played by insomnia and its components, and no data are available for a population of occupational drivers at risk, such as the truck driver category.

**Objective:**

To measure the prevalence and impact of insomnia on motor vehicle accidents (MVAs) and near-miss accidents (NMAs) in 949 truck drivers.

**Design:**

Cross-sectional survey.

**Results:**

Insomnia affected 27.5% of the sample. Compared to other drivers, in the 3 years prior to the study, truck drivers with insomnia had reported a significantly higher number of MVAs; they had also reported a greater number of NMAs in the previous 6 months. After correction for the presence of obstructive sleep apnea, excessive daytime sleepiness, short sleep duration, and other concurrent diseases, insomniac truck drivers had an almost two-fold risk of driving accidents (OR: 1.82, CI 95%:1.33–2.49) and a more than three-fold increased risk of near-miss accidents (OR: 3.35, CI95%:2.06–5.45) compared to non-insomniac drivers.

**Conclusion:**

Insomnia emerged as an independent risk factor for MVAs and NMAs. We strongly advise screening commercial drivers for signs and symptoms of insomnia in order to improve health and safety on the road.

## Introduction

Sleep disturbance and sleep deprivation are highly prevalent in contemporary society with its round-the-clock work schedules. An increasing amount of evidence indicates that sleep habits are crucial factors in the occurrence of occupational and traffic accidents [1–4]. A recently published cross-sectional study revealed that truck drivers have a high prevalence of obstructive sleep apnea (OSA), excessive daytime sleepiness (EDS) and sleep debt, and that each of these three conditions is independently associated with motor vehicle accidents (MVAs) and near miss accidents (NMAs) [5]. In this study, we performed a further data analysis on the same database to determine whether also insomnia plays a role in the occurrence of MVAs and NMAs.

Insomnia disorder is defined in the International Classification of Sleep Disorders, Third Edition [6] as difficulty in initiating or maintaining sleep associated with daytime consequences and not attributable to environmental circumstances or inadequate opportunity to sleep [7]. For the purpose of this study, the term insomnia was used to indicate a disorder in truck drivers who had reported difficulty in falling asleep and/or maintaining sleep, despite adequate opportunity and circumstances for sleep, at least 3 times per week for at least 1 month before our medical examination, and had experienced daytime impairment for this disorder.

According to the ICSD-3 and the Diagnostic and Statistical Manual of Mental Disorders [8], insomnia is associated with impairment of daytime functioning including “proneness for errors or accidents” that has previously been specified as occurring “at work or while driving” [9]. Previous research has provided consistent and convincing evidence of a relationship between EDS, OSA, other sleep disorders and MVAs. However, and somehow surprisingly, very few studies have described the association between insomnia and accidents. Insomnia has been associated with impaired driving performance in laboratory tests [10]. A Norwegian population-based prospective cohort study showed that insomnia is a major contributor to both unintentional fatal injuries in general and fatal motor vehicle injuries [11]. An international cross-sectional survey carried out in 10 countries showed that subjects affected by insomnia had high rates of home accidents, car accidents and work accidents regardless of any adverse effects of hypnotic treatments [12]. In a US national cross-sectional telephone survey, insomnia was associated with 7.2% of all costly workplace accidents and errors and 23.7% of all the costs of these incidents [13]. According to a recent meta-analysis, in insomniac patients, the risk of work injuries is more than double (Odds ratio OR = 2.87) [14]. This figure is similar to the cumulated odds for OSA patients (OR = 2.88). Insomnia and OSA are the two most common sleep disorders, found respectively in 6–20% and 23–50% of the general population [15, 16]. Frequently these disorders are also concurrent, with 39–58% of OSA patients reporting symptoms indicative of co-morbid insomnia [15]. The prevalence of insomniac drivers ranges from 9.3% in regular registered highway car drivers [17] to 26% among older male car drivers who have already had a driving accident [18].

Occupational truck drivers often have to drive long distances and adhere to strict timetables. Consequently, they may pay little attention to fatigue and lack of concentration, thus becoming a population at high risk for road accidents [19]. It has been reported that more than half of truck accidents cause fatal injuries and/or chronic disabilities and that in 80% of cases it is the truck driver who is found to be at fault [20]. As mentioned above, the association of sleep-related problems with accidents has already been investigated in truck drivers, but it appears that only one study carried out on 2,134 truck drivers in Casablanca, in which 36.5% of the participants reported to be insomniac, has taken into account the incidence of insomnia [21]. However, this study failed to test the association of insomnia with accidents. Therefore, to the best of our knowledge this study is the first to investigate the incidence of insomnia in truck drivers and its association with MVAs and NMAs, using a standardized assessment procedure and taking into account the effect of concurrent EDS, short sleep duration (SSD), OSA and other diseases.

## Methods

### Participants and study procedures

This study was part of the "CNH Iveco Industrial Check-Stop Project", an international action for road safety, backed by the UE Road Safety Action and Italian Ministry of Transport. The project was carried out between June 1, 2014 and May 31, 2015. Trained medical staff offered a free medical examination to truck drivers parked in some of the major Italian trucking hubs (Turin, Novara, Verona, Bologna, Rome and Naples). The lack of any relationship between the doctors and the companies employing the drivers guaranteed absolute anonymity. A detailed description of the study method has already been published in this Journal [5]. In that previous work, the topic of insomnia, which is the subject of this study, had not been addressed.

All participants were processed by means of their medical history integrated with questionnaires for sleep disorders and clinical and physical evaluation. A specialist in sleep disorders made the diagnosis of insomnia and any other sleep-related complaints on the basis of both clinical and anamnestic criteria.

The study was approved by the Ethics Committee of the Liguria Region. All participants were informed about the aim of the study and gave written informed consent before participating in the survey.

### Data collection

Data were collected through medical examinations, semi-structured interviews, and standardized questionnaires. During the medical examination, basic socio-demographic characteristics (sex, age, weight, and height), use of tobacco and coffee (number of cigarette packets and cups of coffee per day), and information about any concurrent physical or mental conditions were collected. Respondents were asked about sleeping habits, sleep hygiene, and number of hours of sleep. More specifically, participants were asked to report the number of hours they had actually slept every day in the last 3 months, and the number of hours they wanted to sleep. The occurrence of previous MVAs or NMAs were also investigated, using the following questions: “Have you had a motor vehicle accident at work during the last three years?”, “Have you had a near-miss driving accident during the last six months?”.

Insomnia was assessed with a questionnaire based upon the inclusion criteria of the Research Diagnostic Criteria/International Classification of Sleep Disorder, 3^rd^ Ed. [6] and the DSM-5 [8]. Truck drivers were asked to report whether, for at least 3 nights per week for more than 30 days before the interview they had had: (1) difficulty initiating sleep (more than 30 min to fall asleep) (DIS); (2) difficulty maintaining sleep (DMS); (3) early morning awakening (EMA); (4) non-restorative sleep (NRS), despite adequate opportunity for sleep. Daytime impairment was assessed with questions about the following range of symptoms: fatigue, daytime sleepiness, motivation or energy reduction, tension headaches. Participants were diagnosed as having insomnia if they reported one or more symptoms and at least one form of daytime impairment related to nighttime sleep difficulty.

EDS was evaluated with the Epworth Sleepiness Scale [22]. Diagnosis of suspected OSA was based on clinical examination, integrated with response to the Berlin questionnaire [23]. Participants were also classified as short sleepers if they slept less than 6 hours per night (short sleep duration, SSD), and as very short sleepers if they slept less than 5 hours per night (very short sleep duration, VSSD).

### Statistical analysis

Means and standard deviations were computed for continuous variables, and counts and percentages were computed for categorical variables. Common statistical tests (Student’s t test, Pearson’s chi square) were carried out to compare insomniac and non-insomniac participants.

Logistic regression analysis was used firstly to investigate the univariate association between diagnosis of insomnia and accidents. Odds ratios (OR) and their 95% confidence interval (95%CI) were computed.

Multiple logistic regression analyses, with MVAs and NMAs as response variables, were then carried out to partial out the effect of potential confounding variables. In Model I, background variables (age, smoking status, and coffee consumption) were inserted as further predictors. In Model II, the diagnosis of OSA was also added, while Model III and Model IV included concurrent morbidities and EDS, respectively. Lastly, short sleep duration was also added in Model V.

Statistical analyses were performed with IBM/SPSS 23.0.

## Results

A total of 1,540 truck drivers were contacted and informed of the study; 949 participants completed the questionnaire and the clinical examination (participation rate = 61.6%). All participants were men; age ranged from 18 to 70 years, with a mean age of 44.30 (SD = 10.15). Descriptive statistics of the socio-demographic and sleep characteristics of the sample are summarized in Table 1.

**Table 1:** Demographic characteristics, sleep habits, and symptoms of insomnia.

Variable	Total sample	Insomnia	No-insomnia	*p*	*r*
**Sample size**	949	261 (27.5%)	688 (72.5%)	-	
**Age (years, M±SD)**	44.30±10.16	44.66±10.30	44.16±10.10	n.s.	.02
**BMI (M±SD)**	28.06±4.7	28.18±4.88	28.06±4.66	n.s.	.01
**Coffee consumption (units, M±SD)**	3.20±2.00	3.40±2.30	3.12±1.87	n.s.	.06
**Cigarette consumption (units, M±SD)**	8.13±12.03	8.41±12.00	8.02±12.06	n.s.	.01
**Length of service**	14.0±8.24	17.07±8.47	15.87±7.91	.040	.07
<10 years	198 (20.9%)	44 (16.9%)	154 (22.4%)	n.s.	.06
11–20 years	425 (44.8%)	112 (42.9%)	313 (45.5%)	n.s.	.02
21–30 years	266 (28.0%)	81 (31.5%)	185 (26.9%)	n.s.	.04
>30 years	60 (6.3%)	24 (9.2%)	36 (5.2%)	.028	.07
**Suspect OSA**	245 (25.8%)	109 (41.8%)	136 (19.9%)	< .001	.22
**Concurrent diseases**	409 (43.1%)	141 (54.0%)	268 (39.9%)	< .001	.14
Gastrointestinal diseases	201 (21.2%)	61 (23.4%)	140 (20.3%)	n.s.	.03
Cardiovascular disorders	177 (18.7%)	67 (25.7%)	110 (16.0%)	.001	.11
Diabetes	45 (4.7%)	19 (7.3%)	26 (3.8%)	.023	.07
Depression	87 (9.2%)	36 (13.8%)	51 (7.4%)	.002	.10
Respiratory disorders	76 (8.0%)	30 (11.5%)	46 (6.7%)	.015	.08
Pain conditions	63 (6.6%)	22 (8.4%)	41 (6.0%)	n.s.	.04
**Hours of sleep (done, M±SD)**	6.80±1.45	6.32±1.35	6.98±1.48	< .001	.20
**Hours of sleep (wanted, M±SD)**	7.66±1.57	7.56±1.69	7.71±1.53	n.s.	.04
**Short sleep duration (<6h)**	164 (17.3%)	58 (22.2%)	106 (15.4%)	.013	.08
**Very short sleep duration (<5h)**	44 (4.6%)	20 (7.7%)	24 (3.5%)	.006	.09
**MVA**	330 (34.7%)	126 (48.3%)	204 (29.7%)	< .001	.17
**NMA**	87 (9.2%)	48 (18.4%)	39 (5.7%)	< .001	.20

Note: *p*: p-value; *r*: measure of effect size (*r* < .10: negligible; .10 < *r* < .30: small; .30 < *r* < .50: moderate; *r* > .50: large); *n*: number of participants; *M*: mean; SD: standard deviation; BMI: Body mass index; OSA: Obstructive Sleep Apnea; MVA: motor vehicle accident in the previous 3 years; NM: near miss accident in the previous 6 months.

More than a quarter of the observed population (25.8%) had suspected OSA. The average for sleeping hours was 6.8 h, i.e. less than the recommended number [24, 25].

The prevalence of insomnia was estimated to be 27.5%. As shown in Table 1, insomniac drivers did not differ significantly from non-insomniac drivers in age, BMI, and coffee and tobacco consumption, and the effect size of the significant difference in length of service was negligible (*r* < .10).

The prevalence of OSA was significantly higher in insomniac patients that in non-insomniac drivers, albeit with a small effect size (.10 < *r* < .30). Insomniacs also had a significantly higher prevalence of cardiovascular diseases, diabetes, depression, and respiratory disorders, while the prevalence of gastrointestinal and painful musculoskeletal disorders was similar in the two sub-samples of the population. The insomniac group presented a significantly higher prevalence of SSD and VSSD (22.2% and 7.7% respectively) than the non-insomniac group. As expected, insomniacs slept significantly fewer hours than controls but did not have a greater desire for sleep than the controls. In general, group differences in OSA, concurrent diseases, hours of sleep effected and wanted, and sleep duration never exceeded small in effect size.

Also as expected, small to moderate effect sizes were found when the frequencies of insomnia components were compared. More than one third of participants (330, 34.8%) reported MVAs in the previous 3 years, while 9.2% of the sample reported NMAs in the previous 6 months. The prevalence of both MVAs and NMAs was significantly higher in insomniac drivers than in non-insomniac drivers (Table 1).

In the univariate logistic analyses insomnia was significantly associated with MVAs (OR:2.21, CI95%:1.65–2.99) and NMAs (OR:3.75, CI95%:2.39–5.88).

Multivariate analyses showed that this pattern of results did not change when potential confounders were partialled out (Table 2, S1 and S2 Tables). Correction for OSA, which as we have previously shown [5] was significantly associated with MVAs and NMAs in this sample (Model II), and the inclusion of concurrent diseases (Model III) did not change the observed associations. No change was observed in the level of association between insomnia and accidents after correction for EDS (Model IV) and SSD (<6h) (Model V). Insomniac truck drivers had an almost two-fold risk of MVAs (OR:1.82, CI95%:1.33–2.49) and a more than three-fold increased risk of NMAs (OR: 3.35, CI95%:2.06–5.45) than other drivers.

**Table 2:** Multivariate association between sleep variables, MVAs and NMAs.

	Model I	Model II	Model III	Model IV	Model V
MVAs	OR (95% CI)	OR (95% CI)	OR (95% CI)	OR (95% CI)	OR (95% CI)
**Insomnia**	2.22 (1.65–2.99)***	1.88 (1.38–2.56)***	1.92 (1.41–2.62)***	1.84 (1.34–2.51)***	1.82 (1.33–2.49)***
R^2^	0.046	0.083	0.091	0.095	0.097
**NMAs**					
**Insomnia**	3.67 (2.33–5.78)***	3.12 (1.95–4.98)***	3.32 (2.07–5.33)***	3.27 (2.02–5.30)***	3.35 (2.06–5.45)***
R^2^	0.076	0.096	0.113	0.114	0.118

Model I: corrected for age, coffee consumption, and smoke; Model II: Additionally corrected for OSA; Model III: Additionally corrected for concurrent diseases; Model IV: Additionally corrected for EDS; Model V: Additionally corrected for short sleep duration (<6 h).

*** *p*<0.001

## Discussion and conclusion

Sleep-related problems are known risk factors for road accidents. However, very few studies have investigated the role played by insomnia and its components, and so far no data have been available for a population of occupational drivers at risk such as the truck driver category. This study measured the prevalence and impact of insomnia on MVAs and NMAs in 949 truck drivers. Results revealed that insomnia is very common in truck drivers, as more than 1 out of 4 participants were classified as insomniac, according to DSM V and ICSD III criteria. Moreover, insomnia was shown to be a significant predictor of MVAs and NMAs in this population. The association between insomnia and accidents was not dependent on the concomitant presence of OSA, EDS, SSD, or other diseases.

In this study, the occurrence of insomnia was approximately comparable to the findings of previous epidemiological studies on working populations in which insomnia was diagnosed according to international criteria: 23.6% in a stratified probability sample of US adults [26], 24.0% in a random sample of Chinese industrial workers [27].

The average sleep duration of the whole sample was less than 7 hours, and a non-negligible proportion of drivers reported sleeping less than 6 hours (17.3%), or less than 5 hours a day (4.6%). These data place the participants of this study at the lowest levels on the sleep time scale, if compared to the 2008 telephone survey conducted by the National Sleep Foundation [28], the data collected by the CDC [29], and Australian data relating to the 2016 Sleep Health Foundation National Survey [16]. They are also similar to the data collected by Souza et al. [30] in a sample of Brazilian truck drivers, 50.4% of whom slept between 5 and 6 hours a day. Surprisingly, in this study, insomniac drivers did not manifest a greater desire for sleep than non-insomniac drivers, and the percentage of SSD and VSSD in the insomniac group was significantly higher, but with a negligible effect size. An explanation may be that individuals who are phenotypically capable of regaining strength with a shorter than average sleep duration can cope with insomnia better than those who need more sleep. This could mean that the risk of driving accidents associated with insomnia is higher than the already elevated level estimated in this survey.

In literature, insomnia and sleep duration in workers are both separately and concurrently associated with increased risk of poor work ability [27]. Individuals with insomnia showed a higher rate of workplace accidents than good sleepers [13, 31–33]. In a previous study, insomnia was shown to be significantly associated with workplace and non-workplace injuries among workers without other comorbid conditions; the same association was not demonstrated among workers who were affected by two or more comorbid conditions [26]. In our study, the presence of concurrent morbidity did not significantly affect the association between insomnia and driving accidents.

The high rate of disease in the present sample replicated previous studies on this population. Truck drivers present obesity, hypertension, dyslipidemia, and bad health habits such as smoking, drinking, and insufficient physical exercise, more frequently than the general population [34, 35]. Moreover, various studies have shown that people working shifts or long hours, as truck drivers do, are at higher risk for sleep problems and work injuries [36, 37]. All this evidence indicates that this occupation involves a high risk for workers' health and safety and for the safety of third parties.

In this study we were not able to investigate the relationship between work-related fatigue and insomnia. In industrial workers it has been shown that the effects of insomnia on accident risk can be increased by occupational fatigue [38]. Sustained occupational effort, prolonged hours of work and night shifts, irregular life habit, and exposure to traumatic events, that are very common in commercial drivers, have been associated with sleep disorders in other high-risk occupations such as prison personnel [39] and health care workers [40–42]. In all these occupational examples, researchers observed significant individual vulnerability to performance impairment caused by loss of sleep. In general, however, individuals do not seem to accurately assess the extent of their own vulnerability. A better knowledge of the neurobiological processes of sleep/wake regulation underlying individual variability and the development of methods for identifying workers who are most at risk of sleep loss-related errors and accidents would therefore be helpful in targeting counter measures against fatigue at those subjects who need them the most [43].

To the best of our knowledge, this survey is the largest so far performed on insomnia and accidents in truck drivers. Nevertheless, it has some limitations. Firstly, the cross-sectional nature of the study did not enable us to establish a cause-effect relationship. We can however argue that insomnia is the cause of at least a part of the MVAs or NMAs occurring at the wheel and that the opposite relationship, that is, that driving accidents lead to insomnia, is very unlikely, with the limited exception of cases of post-traumatic stress disorder (PTSD). In our sample, none of the interviewees reported traumatic stress. Secondly, the information on insomnia symptoms and sleep habits was based mainly on self-reported data, integrated with clinical examination. Moreover, due to the self-selection of the participants, only limited generalizability of results was possible. Nevertheless, the non-mandatory nature of the medical examinations allowed drivers to be absolutely sincere with the physicians who did not know them and were not in a position to take any action to restrict their driver’s license. Since the study sample was quite large and contact points were located in the major Italian hubs, we can theorize that no differences would be observed in the population of drivers who daily use the European roads.

In conclusion, our study provides further evidence supporting the need for proper strategies to identify and treat insomnia in truck drivers. Insomnia should be considered a risk factor for accidents regardless of the duration of sleep and the concurrent presence of OSA and other diseases. In 2012 the American College of Occupational and Environmental Medicine’s Task Force on Fatigue Risk Management issued a Guidance Statement which recommended the development of sleep disorder management programs in workplaces, including screening, confirmation, treatment, and compliance, or limited at least to screening for sleep disorders with a questionnaire, possibly combined with a physical examination for risk factors. Employees’ training and education, and responsibility of employees for using the opportunity to sleep appropriately and for obtaining medical care for sleep disorders, are an essential part of these programs [44]. We adhere to this idea and therefore strongly recommend screening commercial drivers for signs and symptoms of insomnia in order to improve health and safety on the road.

## Supporting information

S1 Table Multivariate association between sleep variables and MVAs. (DOCX)

S2 Table Multivariate association between sleep variables and NMAs. (DOCX)
