# Supplementary material for: Insomnia is associated with road accidents. Further evidence from a study on truck drivers
Source: PLoS One. 2017 Oct 31;12(10):e0187256. doi: 10.1371/journal.pone.0187256 (PMC5663450; doi:10.1371/journal.pone.0187256)
Supplement: S1 Table — (DOCX) [file pone.0187256.s001.docx]

**Insomnia and accidents.** **Supporting Information.**

**S1 Table.** Multivariate association between sleep variables and MVAs

|  | **Model I** | **Model II** | **Model III** | **Model IV** | **Model V** |
| --- | --- | --- | --- | --- | --- |
|  | OR (95% CI) | OR (95% CI) | OR (95% CI) | OR (95% CI) | OR (95% CI) |
| **Insomnia** | 2.22 (1.65-2.99)*** | 1.88 (1.38-2.56)*** | 1.92 (1.41-2.62)*** | 1.84 (1.34-2.51)*** | 1.82 (1.33-2.49)*** |
| Age | 0.99 (0.98-100) | 0.99 (0.97-0.99)* | 0.99 (0.97-1.00) | 0.99 (0.97-1.00) | 0.99 (0.97-1.00) |
| Smoke | 0.86 (0.64-1.15) | 0.84 (0.63-1.14) | 0.84 (0.63-1.14) | 0.85 (0.63-1.41) | 0.84 (0.62-1.14) |
| Coffee | 1.08 (0.97-1.19) | 1.06 (0.96-1.18) | 1.06 (0.95-1.17) | 1.05 (0.95-1.17) | 1.05 (0.95-1.17) |
| OSA |  | 2.28 (1.66-3.13)*** | 2.80 (1.95-4.01)*** | 2.70 (1.88-3.89)*** | 2.65 (1.84-3.83)*** |
| Co-morbidity |  |  | 0.66 (0.47-0.93)* | 0.66 (0.47-0.92)* | 0.66 (0.47-0.93)* |
| EDS |  |  |  | 1.43 (0.95-2.14) | 1.43 (0.95-2.15) |
| SSD |  |  |  |  | 1.22 (0.84-1.75) |
| R^2^ | 0.046 | 0.083 | 0.091 | 0.095 | 0.097 |

Model I: corrected for age, coffee consumption, and smoke; Model II: Additionally corrected for OSA; Model III: Additionally corrected for concurrent diseases; Model IV: Additionally corrected for EDS; Model V: Additionally corrected for short sleep duration (<6 h).

* p<0.05; *** *p*<0.001
