# Supplementary material for: Insomnia is associated with road accidents. Further evidence from a study on truck drivers
Source: PLoS One. 2017 Oct 31;12(10):e0187256. doi: 10.1371/journal.pone.0187256 (PMC5663450; doi:10.1371/journal.pone.0187256)
Supplement: S2 Table — (DOCX) [file pone.0187256.s002.docx]

**Insomnia and accidents.** **Supporting Information.**

**S2 Table.** Multivariate association between sleep variables and NMAs

|  | **Model I** | **Model II** | **Model III** | **Model IV** | **Model V** |
| --- | --- | --- | --- | --- | --- |
|  | OR (95% CI) | OR (95% CI) | OR (95% CI) | OR (95% CI) | OR (95% CI) |
| **Insomnia** | 3.67 (2.33-5.78)*** | 3.12 (1.95-4.98)*** | 3.32 (2.07-5.33)*** | 3.27 (2.02-5.30)*** | 3.35 (2.06-5.45)*** |
| Age | 1.00 (0.99-1.02) | 0.99 (0.98-1.02) | 0.99 (0.98-1.02) | 0.99 (0.98-1.02) | 0.99 (0.98-1.02) |
| Smoke | 1.33 (0.82-2.15) | 1.31 (0.81-2.13) | 1.33 (0.81-2.17) | 1.32 (0.81-2.16) | 1.34 (0.82-2.20) |
| Coffee | 0.89 (0.76-1.05) | 0.88 (0.75-1.04) | 0.86 (0.73-1.02) | 0.86 (0.73-1.02) | 0.87 (0.73-1.03) |
| OSA |  | 2.10 (1.30-3.40)*** | 3.17 (1.79-5.62)*** | 3.13 (1.75-5.58)*** | 3.24 (1.81-5.78)*** |
| Co-morbidity |  |  | 0.45 (0.25-0.80)** | 0.45 (0.26-0.81)** | 0.45 (0.25-0.80)** |
| EDS |  |  |  | 1.11 (0.60-2.02) | 1.10 (0.60-2.01) |
| SSD |  |  |  |  | 0.66 (0.35-1.25) |
| R^2^ | 0.076 | 0.096 | 0.113 | 0.114 | 0.118 |

Model I: corrected for age, coffee consumption, and smoke; Model II: Additionally corrected for OSA; Model III: Additionally corrected for concurrent diseases; Model IV: Additionally corrected for EDS; Model V: Additionally corrected for short sleep duration (<6 h).

** p<0.01; *** *p*<0.001
